# Spectroscopic, Physical and Topography of Photochemical Process of PVC Films in the Presence of Schiff Base Metal Complexes

**DOI:** 10.3390/polym8060204

**Published:** 2016-05-25

**Authors:** Emad Yousif, Ali Hasan, Gamal A. El-Hiti

**Affiliations:** 1 Department of Chemistry, College of Science, Al-Nahrain University, Baghdad 64021, Iraq; ali.hasan.89311@gmail.com; 2 Cornea Research Chair, Department of Optometry, College of Applied Medical Sciences, King Saud University, P.O. Box 10219, Riyadh 11433, Saudi Arabia

**Keywords:** poly (vinyl chloride), average molecular weight, quantum yield, UV-Vis spectroscopy, photostability

## Abstract

The photostability of poly(vinyl chloride), PVC, containing various Schiff base metal complexes (0.5% by weight) was investigated. Various indices corresponding to a number of functional groups were monitored with irradiation of polymeric films to determine their photostabilization activities. The quantum yield of the chain scission (Φcs) of modified polymeric films was found to be (1.15–4.65) × 10^6^. The surface morphology of a PVC sample was investigated by the use of atomic force microscope (AFM). The photostability of PVC films in the presence of Schiff base additives was found to follow the following order: PVC < PVC + CuL_2_ < PVC + CdL_2_ < PVC + ZnL_2_ < PVC + SnL_2_ < PVC + NiL_2_. Various mechanisms for PVC films photostability containing the Schiff base additives have been suggested.

## 1. Introduction

Poly(vinyl chloride), PVC, is one of the most consumed materials worldwide [1]. It can be used in various applications, such as packaging, flooring, architecture and electrical wiring [2,3]. However, the thermal and photostability of PVC are low, hindering its use in the field of building construction [4,5]. The poor photostability of PVC by ultraviolet (UV) light (200–700 nm) is presumably due to the formation of carbon-carbon double bonds, carbonyl, hydroperoxide and hydroxy groups within the polymer chains [6]. It was found that photodegradation and photo-crosslinking of PVC led to the release of hydrogen chloride (HCl) and formation polyene polymer along with other side-products but in much lower percentages. A number of environmental problems can be caused by HCl, which is a very corrosive chemical. On the other hand, the presence of chlorine in PVC inhibits aerobic biodegradation [6].

The properties of PVC could be improved by using various ultraviolet absorbers (UVAs) [7,8]. Such additives have the ability to absorb UV light directly. Therefore, they have a tendency to increase stability and, hence, the lifetime of PVC when exposed to sunlight and any other radiation sources [7]. Ultraviolet light stabilizers can be of organic, aromatic, heteroaromatics and inorganic sources. UV absorbers can be used to stabilize a wide range of polymeric materials such as films, plastic and cosmetics. The mechanism by which such additives stabilize PVC involves direct light absorption, peroxide decomposers, radical and/or HCl scavengers [9,10,11,12,13,14,15,16,17,18]. In addition, antioxidants are known as a common photostabilizer, which help in preventing the deterioration of plastics [19]. As part of our own interest in the field of polymers chemistry [20,21,22,23], we have improved the photostability of polymers by using UV photostabilizers [24,25,26,27,28]. Now, we report the improvement in photostability of PVC using Schiff base metal complexes containing 4-(benzylideneamino)-5-(pyridin-4-yl)-4*H*-1,2,4-triazole-3-thiol as new UV-stabilizers.

## 2. Experimental Section

### 2.1. Materials

**Figure 1:**
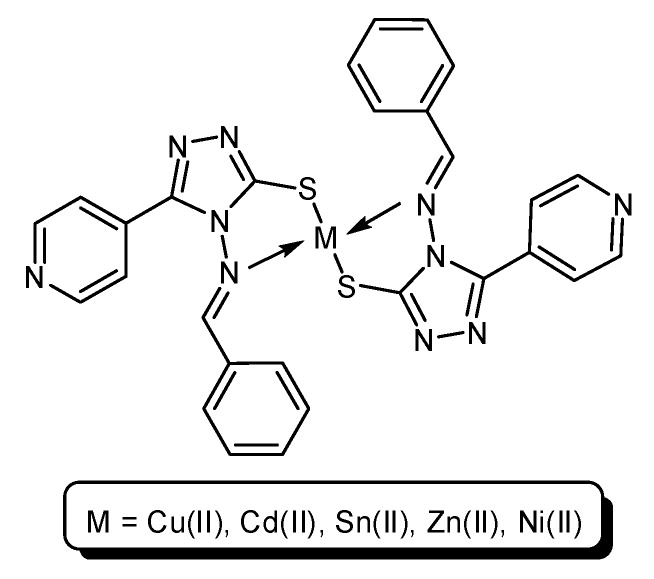
Structure of metal complexes (ML_2_).

Various 4-(benzylideneamino)-5-(pyridin-4-yl)-4*H*-1,2,4-triazole-3-thiol [29] metal complexes (Figure 1) were synthesized according to a reported procedure [30]. The reaction of a mixture containing 4-benzylideneamino-5-(pyridin-4-yl)-4*H*-1,2,4-triazole-3-thiol (two mole equivalents) and various divalent metal salts (one mole equivalent) in boiling ethanol for 1 h gave the corresponding metal complexes. The Schiff base metal complexes obtained were filtered, washed with boiling ethanol and dried. The analytical and spectral data of the synthesized Schiff base metal complexes were in agreement with those reported [30]. The absorption spectra for both ligand and Schiff base complexes were recorded at room temperature in DMSO (10^−3^ M). The ligand showed three absorption bands (263, 307 and 331 nm) in the UV–visible spectrum due to π–π*, π–π* and n–π* electronic transitions, respectively. The Cd(II), Zn(II) and Sn (II) Schiff base complexes show such absorption bands at 265, 306 and 320; 263, 307 and 329; 266, 307 and 319 nm, respectively. A shift to shorter wavelength was observed for the absorption band related to the n–π* electronic transition. The Cu(II) and Ni(II) Schiff bases complexes show similar absorption bands. In addition, they show *d–d* transitions. Ni(II) Schiff bases have complex equilibrium between tetrahedral/square planar (4 coordinates) and octahedral (6 coordinates) geometry [31]. The Ni(II) complex (*T*_d_) shows absorption band at 591 nm which corresponds to ^3^T_1_ (F) → ^3^T_1_ (P) transition [32]. The Cu(II) Schiff base complex (*D*_4h_) shows a band at 494 nm related to ^2^B_1g_ → ^2^A_1g_ transition [33,34]. It should be noted that the difference of coordination atoms is negligible.

### 2.2. Film Preparation

A Digital Vernier Caliper 2610A micrometer (Vogel GmbH, Kevelaer, Germany) was used to prepare PVC films (5 g in 100 mL of tetrahydrofuran, THF; 40 μm) containing Schiff base metal complexes (0.5 wt %) as additives. The films were produced using the evaporation technique (room temperature, 24 h). The PVC polymeric films were dried further, to remove residual THF (room temperature, reduced pressure, 3 h). The transmission of the films should be at least 80%. The PVC films were fixed using aluminum plate stands with a thickness of 0.6 mm (Q-Panel Company, Homestead, FL, USA) [35].

### 2.3. Accelerated Testing Technique

Irradiation of PVC films was carried out by accelerated weather-meter QUV tester (Q-Panel Company, Homestead, FL, USA). A light absorption intensity of 7.75 × 10^−7^ ein·dm^−3^·s^−1^ was used during the photolysis process. The tester equipped with a UV-B 313 fluorescent ultraviolet lamp (40 W) at each side that give 290–360 nm range spectrum in which maximum wavelength was fixed at 313 nm. The polymeric samples were rotated occasionally to ensure that the incident light intensity was the same on all samples [36].

### 2.4. Photodegradation Measuring Methods

#### 2.4.1. Measurement of Polymer Films Photodegradation Rate Using IR Spectrophotometry

Photodegradation of PVC films were monitored using FTIR 8300 Shimadzu Spectrophotometer (400–4000 cm^−1^). The absorption bands for polyene, carbonyl and hydroxyl groups appeared at 1602, 1722 and 3500 cm^−1^, respectively. The progress of photodegradation of PVC can be measured from the changes in the intensity of these absorption bands during the irradiation process. The comparison of these three absorption bands and the peak appeared at 1328 cm^−1^ (reference peak) enables calculation of the polyene (*I*_PO_), carbonyl (*I*_CO_) and hydroxyl (*I*_OH_) indexes [37]. The band index method, Equation (1), was used to calculate the index of such groups (*I*s) from the absorptions of both peak (*As*) and reference (*Ar*). The base line method [37] can be used to calculate the actual absorbance (the difference between top peak and baseline).

(1) Is=As/Ar

#### 2.4.2. Measuring the Photodegradation by Morphology Study

The PVC surface morphology was inspected using atomic force microscopy (AFM), supplied by Veeco (Plainview, NY, USA).

#### 2.4.3. Determination of Average Molecular Weight Using Viscometry Method

The relative molecular weight of PVC films was calculated using the Mark-Houwink relation, Equation (2), and can be measured by Ostwald U-tube viscometer. The average molecular weight (${\bar{M}}_{V}$) is proportional to the intrinsic viscosity, [η], of polymeric materials [38]. Additionally, it is affected by constants (α and *K*) that depend on the polymer-solvent system.

(2) $$\left[\eta \right]=K{\bar{M}}_{V}^{\alpha }$$

Equations (3) and (4) were used to calculate the relative viscosity (η_re_) and specific viscosity (η_sp_) for PVC solution (g/100 mL), respectively, at flow times for pure solvent (*t*_0_) and PVC solution (*t*).

(3) $$\eta_{\text{re}}=\frac{t}{t_{0}}$$

(4) $$\eta_{\text{sp}}=\eta_{\text{re}}-1$$

Equation (5) was used to convert the single-point measurements to the intrinsic viscosities at polymer concentration (*C* = g/100 mL; THF). Molecular weight of PVC films was calculated from intrinsic viscosities.

(5) [η]=(2/C)(ηsp−Inηre)12

The calculation of main chain scission quantum yield (Φ_cs_) is affected by various parameters as shown in Equation (6). These parameters are concentration (*C*), Avogadro’s number (*A*), the initial viscosity–average molecular weight (${\bar{M}}_{V,O}$), incident intensity (*I*_o_) and the irradiation time (*t*) in seconds.

(6) ΦCS=(CA/M¯V,O)⌊([ηo]/[η])1α−1⌋/Iot

## 3. Results and Discussion

### 3.1. FTIR Spectroscopy for PVC Films

**Figure 2:**
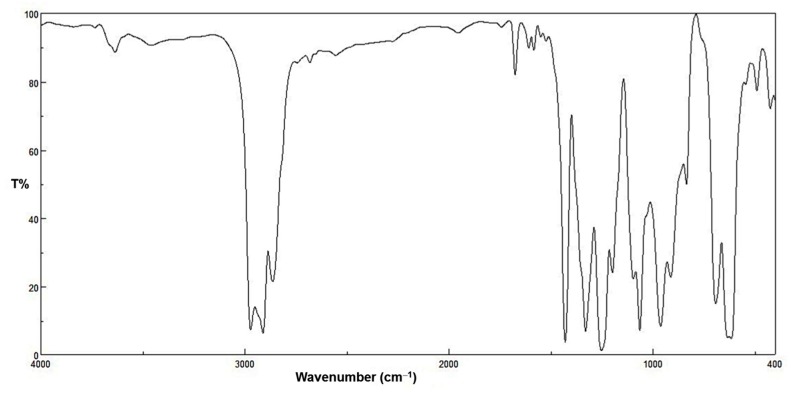
FTIR spectrum of poly(vinyl chloride) (PVC) film before irradiation.

**Figure 3:**
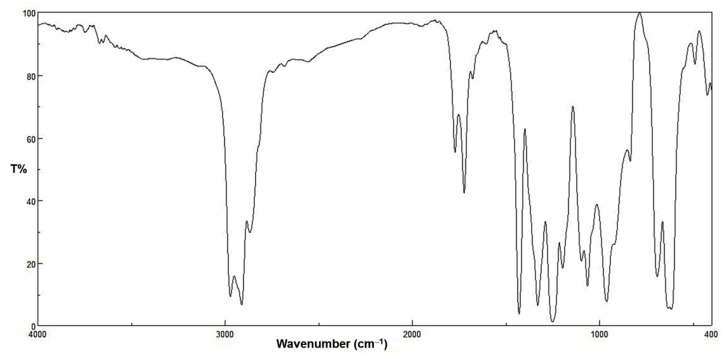
FTIR spectrum of PVC film after irradiation for 250 h.

A series of 4-(benzylideneamino)-5-(pyridin-4-yl)-4*H*-1,2,4-triazole-3-thiol metal complexes were used as additives to improve photostability of PVC. The PVC films were irradiated at λ = 313 nm for 250 h and the changes in FTIR spectra were recorded. The stability of PVC films were studied by recording the changes in polyene, carbonyl, and hydroxyl indices against irradiation time. The bands absorbed at 1602, 1722, and 3500 cm^−1^ were assigned to polyene, carbonyl and hydroxyl groups, respectively, which formed on irradiation of PVC [37]. The FTIR spectra of PVC (control, 40 μm thickness) before and after irradiation for 250 h are shown in Figure 2 and Figure 3, respectively.

**Figure 4:**
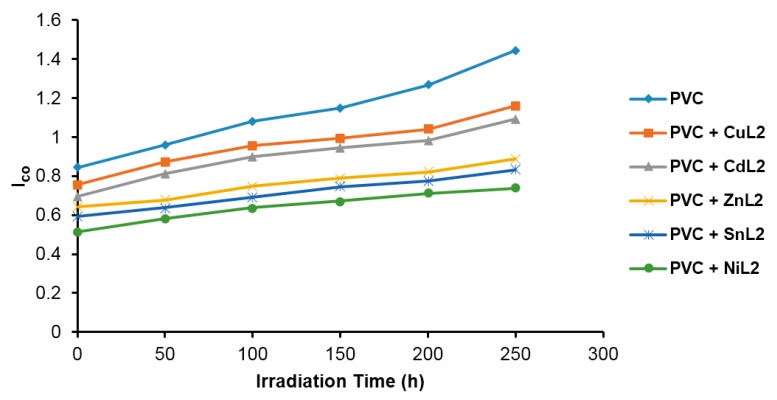
Effect of irradiation time (250 h) on *I*_CO_ for PVC films (40 μm thickness) containing ML_2_ (0.5 wt %).

**Figure 5:**
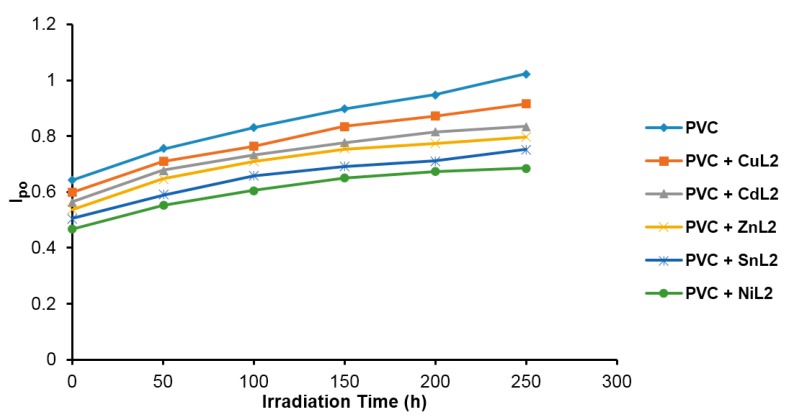
Effect of irradiation time (250 h) on *I*_PO_ and for PVC films (40 μm thickness) containing ML_2_ (0.5 wt %).

**Figure 6:**
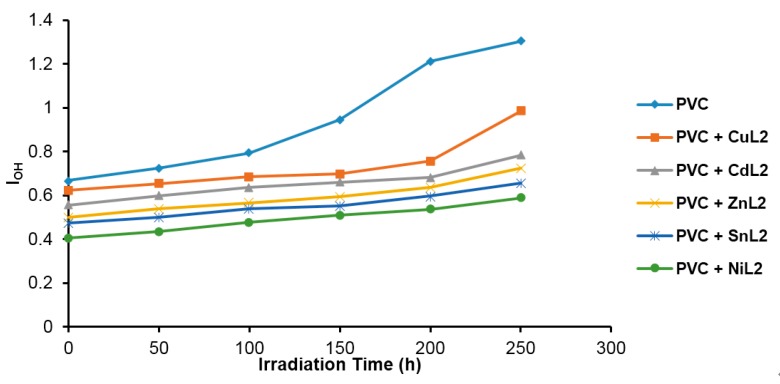
Effect of irradiation time (250 h) on *I*_OH_ and for PVC films (40 μm thickness) containing ML_2_ (0.5 wt %).

The changes in carbonyl (*I*_CO_), hydroxyl (*I*_OH_) and polyene (*I*_PO_) indexes in FTIR spectra of PVC films upon irradiation were monitored (Figure 4, Figure 5 and Figure 6). The changes in carbonyl group starching absorption intensity were used as a confirmation for photodegradation of PVC. Figure 4 shows that the photodegradation of PVC films has been lowered as a result of the addition additive metal complexes (0.5 wt %). Additionally, it shows that the rate of photodegradation of irradiated PVC films was minimum when the metal within additive was Ni(II) followed by Sn(II), Zn(II), Cd(II) and Cu(II) compared to PVC film (control). Therefore, such additives and in particular the one containing Ni(II) can be considered as photostabilizers for PVC polymeric materials.

Figure 5 shows that *I*_PO_ was lower for PVC containing any of the additives compared to PVC itself on irradiation with UV light for up to 250 h. The metal complex containing Ni(II) was the most efficient additive as a PVC photostabilizer and shows a longer induction period compared to the others. The efficiency of the additive containing Sn(II) was next to that of Ni(II) followed by that containing Zn(II), Cd(II) and Cu(II).

Figure 6 shows that *I*_OH_ was higher for PVC (control) compared to PVC containing any of the additives. Again, the additive containing Ni(II) was the most efficient one among others.

### 3.2. Variation of PVC Molecular Weight during Photolysis

**Figure 7:**
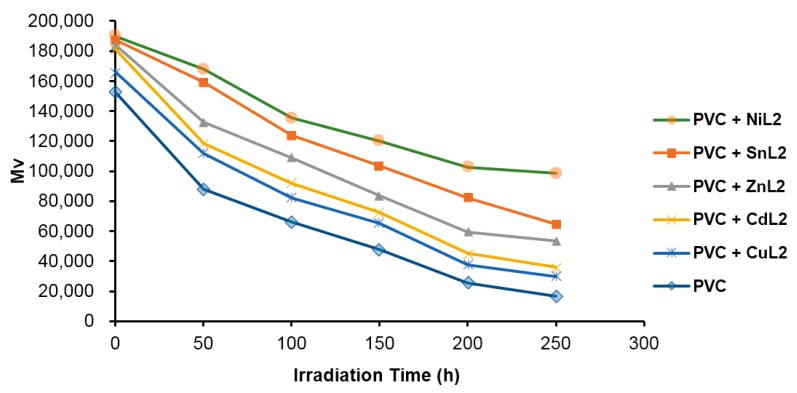
Effect of irradiation time (250 h) on ${\bar{M}}_{V\;}$(g/mol) for PVC films (40 μm thickness) containing ML_2_ (0.5 wt %).

The viscosity average molecular weight $\left({\bar{M}}_{V};\;g/\text{mol}\right)$ of polymeric materials changes as a result of photodegradation [39]. Therefore, the changes in ${\bar{M}}_{V}$ can be used as a test for random chain scission of polymers *versus* irradiation time (250 h) during photolysis process. A light absorption intensity of 7.75 × 10^−7^ ein·dm^−3^·s^−1^ was used during the photolysis process. Figure 7 shows the changes in ${\bar{M}}_{V}$ against irradiation time (250 h) for PVC films (40 μm thickness) containing ML_2_ (0.5 wt %) and that of PVC itself (control).

**Figure 8:**
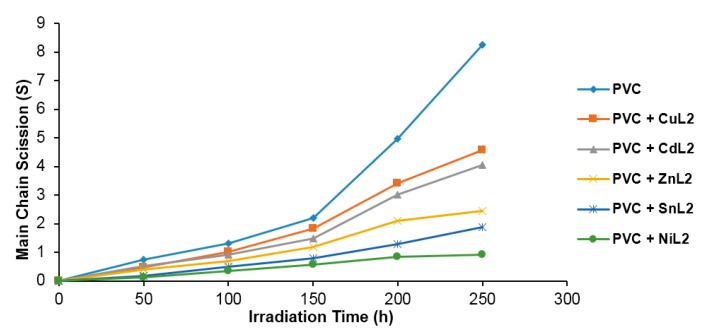
Effect of irradiation time (250 h) on *S* for PVC films (40 μm thickness) containing ML_2_ (0.5 wt %).

${\bar{M}}_{V}$ was measured in THF at room temperature using Equation (3). There were some insoluble PVC traces which are an indication for branching or cross-linking that might have taken place during photolysis process [40]. To confirm that, the number of average number cut per single chain (average chain scission; *S*) *versus* irradiation time (*t*) was calculated using Equation (7) [41].

(7) $$S={\bar{M}}_{V,O}/{\bar{M}}_{V,t}-1$$

where ${\bar{M}}_{V,O}\;$is the viscosity average molecular weight before irradiation and ${\bar{M}}_{V,t}\;$is the viscosity average molecular weight at irradiation time *t*. The changes in average chain scissions (*S*) *versus* irradiation time are presented in Figure 8. It indicates that cross-linking within the polymeric chains led to an increase in branching proportion. Some insoluble residues were formed during the irradiation process.

Weak bonds that are randomly distributed within polymeric chains can be cleavage quickly in the initial stages of photodegradation process [42,43]. The degree of deterioration (α) can be calculated from the initial molecular weight (*m*), average chain scission (*S*) and ${\bar{M}}_{V}$ (Equation (8)).

(8) $$\alpha =m\times S/{\bar{M}}_{V}$$

**Figure 9:**
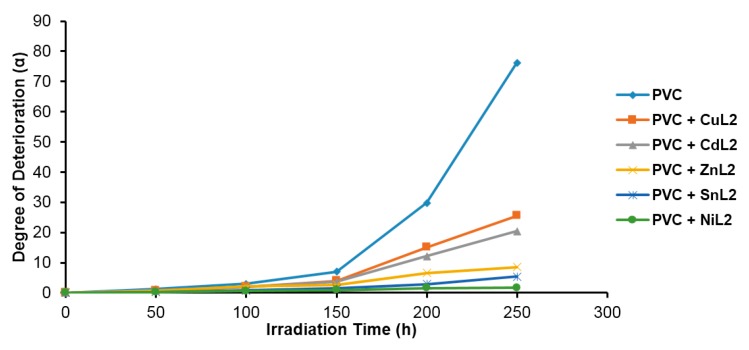
Effect of irradiation time (250 h) on α for PVC films (40 μm thickness) containing ML_2_ (0.5 wt %).

Figure 9 shows the effect of irradiation time (up to 250 h) of PVC films on the degree of deterioration (α). There was a sharp and rapid increase in the α values when the irradiation time was 150 h. This could be an indication of a random bond cleavage within polymeric chains. The changes in α values for the irritated PVC (control) were higher and sharper compared to those obtained from PVC films containing additives. Clearly, the additive-containing Ni(II) shows the minimum change in α values and can improve the photostabilization of PVC significantly. The efficiency of additives follows the order of PVC + NiL_2_, PVC + SnL_2_, PVC + ZnL_2_, PVC + CdL_2_ and PVC + CuL_2_.

The chain scission quantum yield (Φ_cs_) can be used to provide additional proof for the photodegradation process [44]. The Φ_cs_ for PVC films was calculated using Equation (6) and reported in Table 1. The Φ_cs_ for pure PVC was of course higher than those of PVC + ML_2_. The Φ_cs_ value was lowest in PVC + NiL_2_ and follow the order of PVC + NiL_2_ < PVC + SnL_2_ < PVC + ZnL_2_ < PVC + CdL_2_ < PVC + CuL_2_. The low Φ_cs_ for PVC in the presence of any additives was predicated [25]. It seems that the irradiation energy was absorbed at one site within the PVC macromolecule followed by electronic excitation that was distributed over a number of bonds, which makes a single bond breaking low. Non-reactive processes might be also involved, which led to dissipation of the absorbed energy.

### 3.3. Surface Analysis

**Figure 10:**
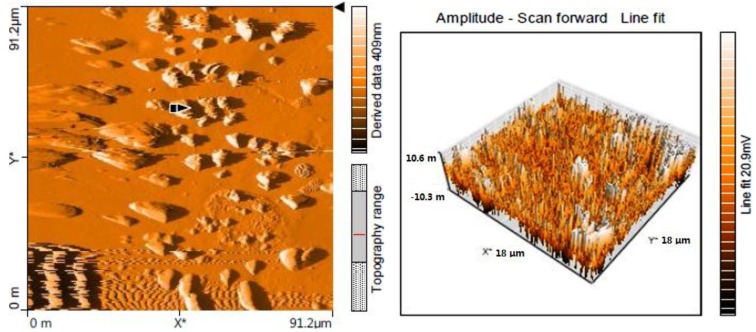
2D and 3D AFM images of PVC film (control) exposed to UV light for 250 h.

**Figure 11:**
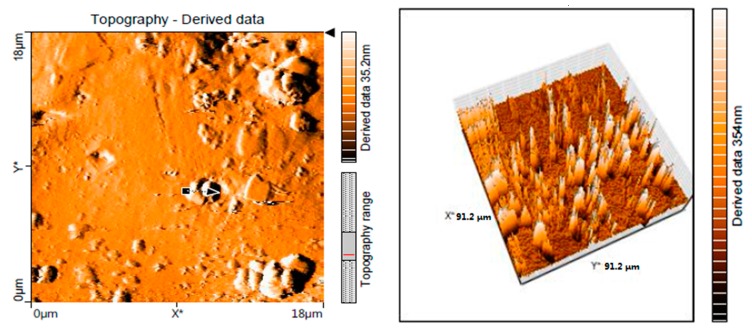
2D and 3D AFM images of PVC film containing Ni(II) exposed to UV light for 250 h.

An atomic force microscope (AFM) was used to study the PVC polymeric films’ morphology. Such a technique is an excellent tool to measure the roughness factor (*R*q), pore size and two- and three-dimensional topographic images of PVC films. The AFM technique was used over a scanned area of 5.0 × 5.0 μm^2^. The PVC films, in the presence of additive containing Ni(II) and the controlled PVC, after irradiation with UV light for 250 h were subjected to AFM (Figure 10 and Figure 11, respectively).

The surface of irradiated PVC film containing Ni(II) was very smooth (*R*q = 34.8 nm) compared to the controlled PVC film. The AFM images for the PVC film (control) showed a highly rough surface (*R*q = 116.3). This rough surface could be explained as a result of bonds breaking after exposure to UV light and removal of leachable from the polymer surface [45]. The roughness within the PVC (blank) surface could be due dehydrochlorination on irradiation. It has been reported that PVC dehydrochlorination can take place at 150 °C [46]. Clearly, the metals in the Schiff base complexes could inhibit such processes.

### 3.4. Suggested Mechanisms of PVC Photostabilization in the Presence of Additives

**Scheme 1:**
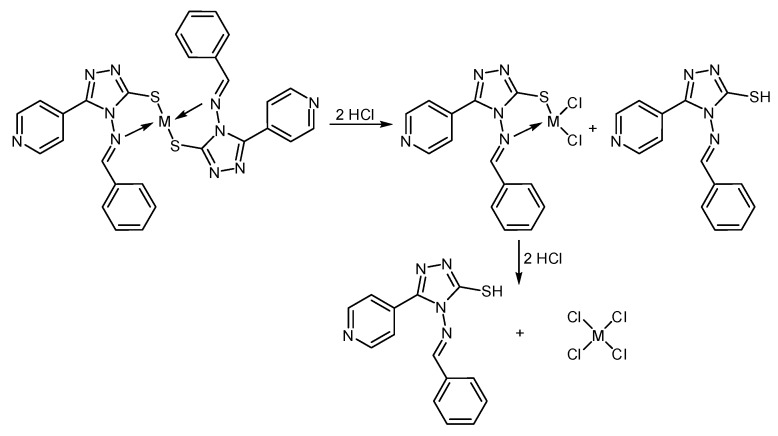
Additives act as HCl scavengers.

**Scheme 2:**
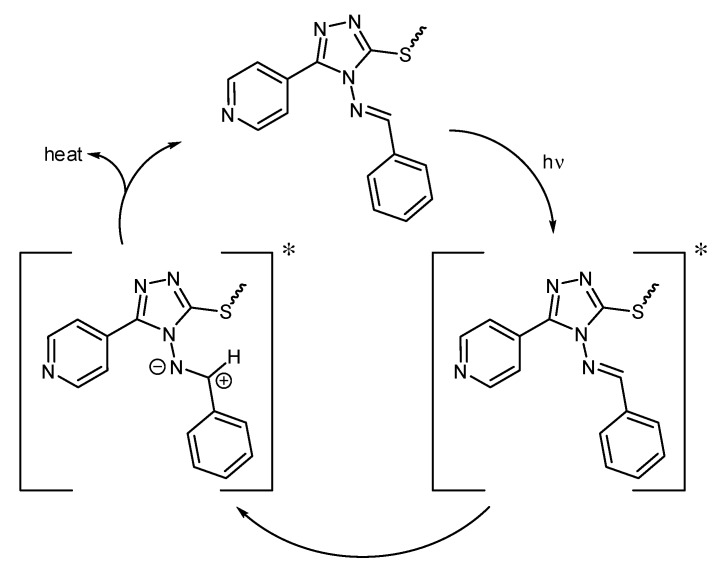
Additives act as UV Absorbers.

**Scheme 3:**
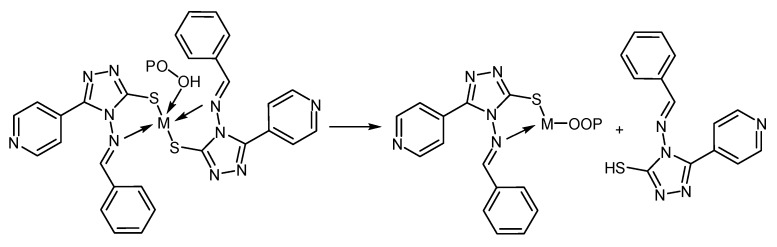
Additives as peroxide decomposers.

**Scheme 4:**
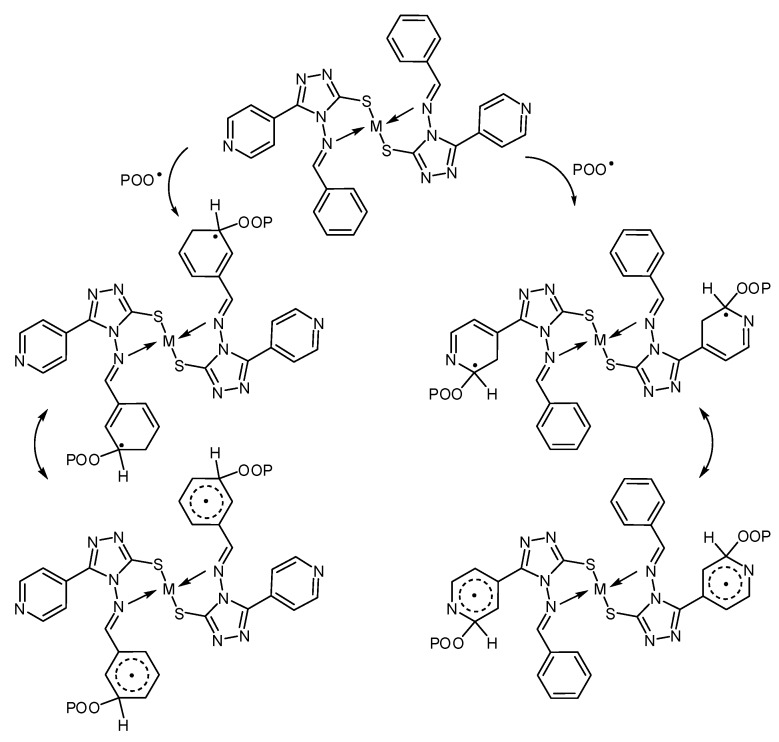
Additives act as radical scavengers.

The efficiency of additives as PVC photostabilizers was measured as a function of the changes in the concentration of carbonyl, polyene and hydroxyl groups. The changes were much smaller in PVC films containing Schiff base metal complexes compared to PVC itself. Various mechanisms for the photostabilization process of PVC samples containing such additives can be suggested (Scheme 1, Scheme 2, Scheme 3 and Scheme 4). Metal cations, such as Ni(II), Sn(II), Zn(II) and Cd(II), are strong Lewis acids that can scavenge HCl (Scheme 1) and known as secondary stabilizers. Therefore, Schiff base additives containing such metals are expected to provide photostability for PVC films for long period. Additionally, it has been reported that thiols can act as heat stabilizers and increase the stability of PVC [47,48]. The stabilization effect could be due the displacement of PVC chlorine atoms by thiol sulfur atoms and formation of sulfur carbon bonds along with the generation of HCl.

Aromatic and heteroaromatic rings can act as UV absorbers [49]. Such electron rich systems within additive backbone structures can absorb the UV radiation energy directly and dissipates it to harmless heat energy (Scheme 2).

Polymeric materials can undergo degradation in the presence of hydroperoxides through photooxidation. The addition of UV photostabilizer additives is known to protect the polymeric films from photodegradation [50]. The Schiff base metal complexes are expected to decompose the peroxide and quench the excited state and lead to stabilization of PVC films (Scheme 3).

The additives could also stabilize the PVC samples through acting as radical scavengers in the presence of a chromophore (POO). The energy can be transferred through formation of a complex between the chromophore, in excited state, and additive containing metal. Such unreactive charge transfer complexes could be stabilized via resonance of aromatic rings within the additives (Scheme 4). Photostabilization process could be also explained due to possible attraction between polarized C−N bonds in pyridine and triazole rings within additives and PVC polymeric chains. Such attraction can play a role to allow the energy of excited PVC films to be transferred effectively to the additives and then to a harmless energy.

## 4. Conclusions

The photostabilization of PVC films using 4-(benzylideneamino)-5-(pyridin-4-yl)-4*H*-1,2,4-triazole-3-thiol metal complexes was successfully achieved. Photostabilization of PVC films containing additives showed a decrease in carbonyl, polyene and hydroxyl indices in order of CuL_2_ > CdL_2_ > ZnL_2_ > SnL_2_ > NiL_2_. The additives could act as HCl scavengers, UV absorbers, peroxide decomposer and/or radical scavengers and therefore stabilize the PVC films. The Ni(II) complex was the most efficient additive in photostabilization of PVC containing Schiff’s base metal complexes. For long term use of such additives, the possible problems that could arise from the migration and/or toxicity of such complexes need to be investigated.

## Figures and Tables

**Table 1:** The chain scission quantum yield (Φ_cs_) for PVC films after irradiation. ^a^

PVC Film (0.5 wt %)	Quantum Yield of Main Chain Scission (Φ_cs_)
PVC	4.65 × 10^−6^
PVC + CuL_2_	2.19 × 10^−6^
PVC + CdL_2_	8.69 × 10^−7^
PVC + ZnL_2_	4.19 × 10^−7^
PVC + SnL_2_	1.93 × 10^−7^
PVC + NiL_2_	1.15 × 10^−7^

^a^ PVC films of 40 μm thickness, irradiation for 250 h.

## References

[1] Saeki Y., Emura T. (2002). Technical progresses for PVC production. Prog. Polym. Sci..

[2] Zhang X., Zhao T., Pi H., Guo S. (2010). Mechanochemical preparation of a novel polymeric photostabilizer for poly(vinyl chloride). J. Appl. Polym. Sci..

[3] Real L.E.P., Ferraria A.M., Botelho de Rego A.M. (2008). Comparison of different photo-oxidation conditions of poly(vinyl chloride) for outdoor applications. Polym. Test..

[4] Nicholson J.W. (2012). The Chemistry of Polymers.

[5] Decker C., Owen E.D. (1984). Degradation and Stabilisation of PVC.

[6] Gewert B., Plassmann M.M., MacLeod M. (2015). Pathways for degradation of plastic polymers floating in the marine environment. Environ. Sci. Process. Impact..

[7] Blanchet T., Olabisi O. (1997). Handbook of Thermoplastics.

[8] Yoshioka T., Kameda T., Ieshige M., Okuwaki A. (2008). Dechlorination behaviour of flexible poly(vinyl chloride) in NaOH/EG solution. Polym. Degrad. Stab..

[9] Shih H.-K., Chen Y.H., Chu Y.L., Cheng C.-C., Chang F.-C., Zhu C.-Y., Kuo S.-W. (2015). Photo-crosslinking of pendent uracil units provides supramolecular hole injection/transport conducting polymers for highly efficient light-emitting diodes. Polymers.

[10] Griffini G., Bella F., Nisic F., Dragonetti C., Roberto D., Levi M., Bongiovanni R., Turri S. (2015). Multifunctional luminescent down shifting fluoropolymer coatings: A straightforward strategy to improve the UV-Light harvesting ability and long term outdoor stability of organic dye sensitized solar cells. Adv. Energy Mater..

[11] Bella F., Griffini G., Gerosa M., Turri S., Bongiovanni R. (2015). Performance and stability improvements for dye-Sensitized solar cells in the presence of luminescent coatings. J. Power Sour..

[12] Fouassier J.P., Lalevée J. (2014). Photochemical production of interpenetrating polymer networks; simultaneous initiation of radical and cationic polymerization reactions. Polymers.

[13] Mosnáček J., Kundys A., Andicsová A. (2014). Reversible-deactivation radical polymerization of methyl methacrylate induced by photochemical reduction of various copper catalysts. Polymers.

[14] Yousif E., Haddad R. (2013). Photodegradation and photostabilization of polymers, especially polystyrene: Review. SpringerPlus.

[15] Buruaga L., Pomposo J.A. (2011). Metal-Free polymethyl methacrylate (PMMA) nanoparticles by enamine “click” chemistry at room temperature. Polymers.

[16] Ratnam C.T. (2001). Irradiation crosslinking of PVC-ENR blend effect of efficient radical scavenger. Plast. Rubber Compos..

[17] Wypych G. (2008). PVC Degradation and Stabilization.

[18] Andrady A.L., Hamid S.H., Hu X., Torikai A. (1998). Effects of increased solar ultraviolet radiation on materials. J. Photochem. Photobiol. B.

[19] Chmela Š., Lajoie P., Hrdlovič P., Lacoste J. (2000). Combined oligomeric light and heat stabilizers. Polym. Degrad. Stab..

[20] Smith K., El-Hiti G.A., Al-Zuhairi A.J. (2011). The synthesis of polymeric sulfides by reaction of dihaloalkanes with sodium sulphide. J. Sulfur Chem..

[21] Smith K., Balakit A.A., Pardasani R.T., El-Hiti G.A. (2011). New polymeric sulfide–borane complexes: Convenient hydroborating and reducing reagents. J. Sulfur Chem..

[22] Smith K., Balakit A.A., El-Hiti G.A. (2012). Poly(propylene sulfide)-borane: Convenient and versatile reagent for organic synthesis. Tetrahedron.

[23] Smith K., Al-Zuhairi A.J., El-Hiti G.A., Alshammari M.B. (2015). Comparison of cyclic and polymeric disulfides as catalysts for the regioselective chlorination of phenols. J. Sulfur Chem..

[24] Yousif E., El-Hiti G.A., Hussain Z., Altaie A. (2015). Viscoelastic, spectroscopic and microscopic study of the photo irradiation effect on the stability of PVC in the presence of sulfamethoxazole Schiff’s bases. Polymers.

[25] Balakit A.A., Ahmed A., El-Hiti G.A., Smith K., Yousif E. (2015). Synthesis of new thiophene derivatives and their use as photostabilizers for rigid poly(vinyl chloride). Int. J. Polym. Sci..

[26] Yousif E., El-Hiti G.A., Haddad R., Balakit A.A. (2015). Photochemical stability and photostabilizing efficiency of poly(methyl methacrylate) based on 2-(6-methoxynaphthalen-2-yl)propanoate metal ion complexes. Polymers.

[27] Yousif E., Haddad R., Ameer A.B., Win Y.-F. (2014). Ultra-violet spectra studies of photodegradation of PVC films in presence of Fe(III) chelate complex. Eur. J. Chem..

[28] Yousif E., Abdallh M., Hashim H., Salih N., Salimon J., Abdullah B.M., Win Y.-F. (2013). Optical properties of pure and modified poly(vinyl chloride). Int. J. Ind. Chem..

[29] Koparir M., Koparir P., Cansiz A., Temuz M.M. (2010). Synthesis of some 5-substituted-1,2,4-triazole-3-thiones, containing thiourea, arylidenamino and morpholin-4-yl methyl fragments. Asian J. Chem..

[30] Hasan A., Ameer A., Ahmed A., Yousif E. (2015). Synthesis and characterization of some transition metal (II) complexes with 1,2,4-triazole Schiff base. J. Chem. Pharm. Res..

[31] Kwiatkowski E., Kwiatkowski M. (1980). Unsymmetrical Schiff base complexes of nickel(II) and palladium(II). Inorg. Chim. Acta.

[32] Joseyphus R.S., Nair M.S. (2010). Synthesis, characterization and biological studies of some Co(II), Ni(II) and Cu(II) complexes derived from indole-3-carboxaldehyde and glycylglycine as Schiff base ligand. Arab. J. Chem..

[33] Akila E., Usharani M., Ramachandran S., Jayaseelan P., Velraj G., Rajavel R. (2013). Tetradentate-arm Schiff base derived from the condensation reaction of 3,30-dihydroxybenzidine, glyoxal/diacetyl and 2-aminophenol: Designing, structural elucidation and properties of their binuclear metal(II) complexes. Arab. J. Chem..

[34] Osowole A.A. (2011). Synthesis, characterization, and magnetic and thermal studies on some metal(II) thiophenyl Schiff base complexes. Int. J. Inorg. Chem..

[35] Shneshil M.K., Redayan M.A. (2011). Photostabilization of PVC films by using some novel tetra Schiff’s bases derived from1,2,4,5-tetra-[5-amino-1,3,4-thiadiazole-2-yl]benzene. Diyala J. Pure Sci..

[36] Yousif E.A., Hameed A.S., Bakir E.T. (2007). Synthesis and photochemical study of poly(vinyl chloride)-1,3,4-oxadiazole and 1,3,4-thiadiazole. J. Al-Nahrain Univ..

[37] Rabek J., Ranby B. (1975). Photodegradation, Photooxidation and Photostabilization of Polymers.

[38] Mark J. (2007). Physical Properties of Polymers Handbook.

[39] Geuskens G., Compton R.G., Bamford C.H., Tipper C.F.H. (1975). Photodegradation of polymers. Comprehensive Chemical Kinetics.

[40] Mori F., Koyama M., Oki Y. (1977). Studies on photodegradation of poly(vinyl chloride (part I). Die Angew. Makromol. Chem..

[41] Scott G. (1973). Polymers and Ecological Problems.

[42] Gugumus F. (1990). Mechanism of Polymer Degradation and Stabilization.

[43] Shyichuk A.V., White J.R. (2000). Analysis of chain-scission and crosslinking rates on the photooxidation of polystyrene. J. Appl. Polym. Sci..

[44] Yousif E.A., Aliwi S.M., Ameer A.A., Ukal J.R. (2009). Improved photostability of PVC films in the presence of 2-thioacetic acid-5-phenyl-1,3,4-oxadiazole complexes. Turk. J. Chem..

[45] Kara F., Aksoy E., Yuksekdagd Z., Hasirci N., Aksoy S. (2014). Synthesis and surface modification of polyurethanes with chitosan forantibacterial properties. Carbohydr. Polym..

[46] Zheng X.-G., Tang L.-H., Zhang N., Gao Q.-H., Zhang C.-F., Zhu Z.-B. (2003). Dehydrochlorination of PVC materials at high temperature. Energ. Fuels.

[47] Starnes W.H., Du B., Kim S., Zaikov V.G., Ge X., Culyba E.K. (2006). Thermal stabilization and plasticization of poly(vinyl chloride) by ester thiols: Update and current status. Thermochim. Acta.

[48] Folarin O.M., Sadiku E.R. (2011). Thermal stabilizers for poly(vinyl chloride): A review. Int. J. Phys. Sci..

[49] Yousif E., Bakir E., Salimon J., Salih N. (2012). Evaluation of Schiff bases of 2,5-dimercapto-1,3,4-thiadiazole as photostabilizer for poly(methyl methacrylate). J. Saudi Chem. Soc..

[50] Pospíšil J., Klemchuk P.P. (1989). Oxidation Inhibition in Organic Materials.

